# A letter to the editor: reflection on medical student volunteer role during the coronavirus pandemic

**DOI:** 10.1080/10872981.2020.1784373

**Published:** 2020-06-29

**Authors:** Ariella Levene, Ciara Dinneen

**Affiliations:** Bristol Medical School, Bristol, UK

**Keywords:** Medical student volunteer, coronavirus, COVID-19

Due to the coronavirus pandemic causing an abrupt end to clinical teaching, medical students all over the country suddenly found themselves with little to do and mostly heading back to their family homes, scattered all over the country. We were two of these medical students. Based usually in Bristol we found ourselves in London, the ‘epicentre’, with news of our local hospitals becoming increasingly overwhelmed with COVID-19 patients. We were eager to help and put our studies to use. On the 6^th^ of April we both began working in ITU which was starting to operate at double its usual capacity. Our jobs included reorganising surgical wards to make way for more ITU beds, mask fit testing and PPE training ITU staff, proning COVID-19 patients and facilitating video calls between patients and families who could not visit due to the new restrictions.

Joining the proning team was our first introduction to the large repurposed COVID-19 wards which were overfilled with ventilated patients and also allowed us to begin adjusting to the hours we would spend in PPE. Despite this solemn introduction to working for the NHS, for the first time we were relied upon as part of a team made up of a variety of medical professionals including surgeons, physiotherapists and other medical students who had been thrown together to help out. It was, however, the last job facilitating the video calls between families and ITU patients which had the most profound and lasting impact on us.

As medical students, this was the first time we had the responsibility of contacting a patient’s family. It was clear to us that the families on the other end of the line were frightened and distressed [1], even more so due to the context of the pandemic and inability to visit. The importance of providing a daily video call for these patients and their families was immediately evident. We experienced many unforgettable highs and lows. Within the first week we helped facilitate an end of life call for a patient’s family. This was the first time we experienced a family taking emotional support directly from us, as part of the medical team [2]. Alongside the very sad, we also experienced the unwavering support and pure joy shared during these calls. We witnessed patients talking after weeks of frustration filled silence, patients walking for the first time and patients being discharged. We celebrated these achievements together with the patient and their families. It was a great privilege.

Thinking ahead to how the learning we have been fortunate to receive during this crisis could be captured outside of a pandemic for other medical students, we reflect on the enormous value of actually working to support our healthcare colleagues. We ask ourselves would it be possible to re-create this role during ordinary times. There is so much for us to learn from dedicating more of our time to understanding the roles of other healthcare professionals and working alongside to support them. In addition to this, working so closely with students from many different medical schools has been an invaluable learning experience. An emphasis on this going forward in the medical curriculum could open doors to new resources and ways of learning. This pandemic has shone a light on many opportunities to enhance the medical curriculum. We both know that the experience we had during this crisis will shape the doctors we hope to become.

Just a few months ago, while on our ITU placements back in Bristol there was no way we could have imagined that we would be working to support frontline staff in the midst of a global pandemic. We put ourselves outside of our comfort zones and learned a great deal in the process.
